# Intermittent Fasting—Short- and Long-Term Quality of Life, Fatigue, and Safety in Healthy Volunteers: A Prospective, Clinical Trial

**DOI:** 10.3390/nu14194216

**Published:** 2022-10-10

**Authors:** Katharina Anic, Mona W. Schmidt, Larissa Furtado, Lina Weidenbach, Marco J. Battista, Marcus Schmidt, Roxana Schwab, Walburgis Brenner, Christian Ruckes, Johannes Lotz, Karl J. Lackner, Annbalou Hasenburg, Annette Hasenburg

**Affiliations:** 1 Department of Gynecology and Obstetrics, University Medical Center of Johannes Gutenberg, University Mainz, 55131 Mainz, Germany; 2 Interdisciplinary Center Clinical Trials, University Medical Center Mainz, 55131 Mainz, Germany; 3 Institute of Clinical Chemistry and Laboratory Medicine, University Medical Center of Johannes Gutenberg University Mainz, 55131 Mainz, Germany; 4 Charite University Hospital Berlin, 10117 Berlin, Germany

**Keywords:** intermittent fasting, quality of life, diet, Fatigue Assessment Scale (FAS), chronic pain

## Abstract

Background: Intermittent fasting (IF) is defined as an eating pattern without calorie restrictions, alternating between periods of fasting and eating. In the past decades IF has not only become a popular weight-reducing diet but is thought to improve Quality of Life (QoL) and fatigue. However, very little evidence exists for the general population. Thus, we aimed to assess the impact of a 16-h fasting period per day over a three-month study period on QoL and especially fatigue in healthy people. Methods: We conducted a prospective cohort study including healthy subjects. All participants fasted 16 h for at least five days a week while maintaining their normal lifestyle. In the study, we analysed blood samples as well as QoL through standardized questionnaires (WHO-5 questionnaire, Short Form Health 36). Furthermore, we measured the degree of fatigue with the Fatigue Assessment Scale (FAS) and Fatigue Severity Scale (FSS) as well as compliance, activity records, and weight alterations. All endpoints were evaluated at baseline, after two weeks, four weeks, and three months of IF. Results: A total of 30 participants fasted for the entire study period. The results of the WHO-5 questionnaire (15.6 ± 4.6 vs. 18 ± 3.6, *p* < 0.0019) demonstrated a significant increase in QoL. For long-term QoL six out of eight domains measured by the Short Form Health 36 (SF-36) significantly improved (e.g., physical health: 92.3 ± 11.6 vs. 96.5 ± 6.3, *p* = 0.015; mental health: 75.5 ± 12.0 vs. 81.7 ± 9.0; *p* < 0.001 and body pain: 74.1 ± 31.8 vs. 89.5 ± 14.9; *p* = 0.008) after three months. Fatigue significantly decreased from 10.3 ± 3.2 to 8.4 ± 2.5; *p* = 0.002 for mental fatigue and from 12.6 ± 3.8 to 10.7 ± 3.3; *p* = 0.002 measured by the FAS. The mean FSS-Score at baseline was 3.5 ± 1.2 compared to 2.9 ± 1.1 (scale 1–7) after three months (*p* < 0.001). Notably, the proliferation marker IGF-1 was significantly reduced. No clinically significant changes in laboratory parameters were observed that would have endangered a participant’s safety. Conclusions: IF according to the 16:8 regime over a fasting period of three months significantly improved several aspects of the QoL and decreased fatigue in healthy people, while maintaining a good safety profile. The practicability of this diet was also demonstrated for shift workers and people with a high percentage of active labour. Apart from the improvement in QoL and fatigue, the significant reduction in IGF-1, which can act as an accelerator of tumour development and progression, might be an indicator of the potential benefits of IF for patients with cancer.

## 1. Introduction

Intermittent fasting (IF) has gained popularity as a health-promoting diet [1]. It started in the late 19th century with experiments on the lifespan of rodents under the condition of IF. Moreover, it had already been a traditional part of the Islamic culture (i.e., Ramadan) long before that [2,3]. In 2020, IF became the most popular diet in the United States according to the International Foundation for Integrated Care [4]. While the term “intermittent fasting” describes a specific type of time-restricted eating (also referred to as cyclic fasting or fasting-mimicking diet), multiple versions following a common regimen exist: For a certain period of time, no food may be consumed [5]. This period alternates with a usually shorter interval when food intake is unrestricted. The interval of food deprivation can vary from 12 h to several days [6].

The suggested benefits of IF are based on the concept of inducing a switch from an anabolic metabolism to a fasting metabolism, where greater proportions of the required energy are gained from ketone bodies [7]. Ketone bodies including acetone, acetoacetate, and β-hydroxybutyrate are produced in larger amounts, once blood sugar levels drop and adrenalin and glucagon levels rise after a few hours of food deprivation [6]. Their synthesis from free fatty acids occurs simultaneously with increased gluconeogenesis in the liver [8,9]. In current hypotheses, this metabolic switch does not only promote weight loss, but also positively influences chronic diseases such as the metabolic syndrome [5,10,11,12] or neurologic diseases [5,10,13,14,15]. Moreover, IF has demonstrated a reduction in oxidative stress markers [10], which play an important role in many diseases, such as the progression of endometriosis [16]. Endometriosis affects up to 10% of all premenopausal women [17,18] and the dispersed endometrial cells can lead to an inflammatory reaction as well as symptoms such as severe premenstrual and perimenstrual pain [19,20], which in turn reduce Quality of Life (QoL), cause mental health problems, and promote depression [21].

Furthermore, Quality of Life (QoL) and fatigue are often especially impaired in cancer patients undergoing cytotoxic chemotherapy. In long-term studies, IF has not only been shown to inhibit tumour development and cancer progression, but also to reduce side effects in patients treated with chemotherapy [3,22,23]. Short-term fasting during chemotherapy has demonstrated positive effects on fatigue and quality of life (QoL) in the current literature [24,25,26]. However, in these trials, a few short-term continuous fasting periods of 48 to 72 h were used, rather than an actual diet modification [24,27]. IGF-1 plays a critical role in tumour development and progression. By lowering the activation of the mTOR signalling pathway through reduced IGF-1 levels, the mTOR regulated inhibition of autophagy is reduced. Thus, more proteins are reused instead of being synthesized and healthy cells become more resistant to oxidative stress. 

With an increasingly obese and aging population, many health-related concerns exist despite not yet needing active treatment. While there is some evidence on IF in specific patient populations or cultural fasting [28,29,30], very little is known about the actual health and QoL benefits in a mixed, generally healthy study population. Thus, in this prospective clinical trial, we aimed to assess the potential of IF for increasing the QoL in overall healthy people, as well as its safety and implementation in everyday life.

## 2. Materials and Methods

### 2.1. Study Design and Setting

This prospective cohort study was conducted in the Department of Gynecology and Obstetrics of the University Medical Centre of the Johannes Gutenberg University, Mainz, Germany.

A heterogeneous group of adults with no specific health profile was recruited to adhere to an IF diet following the 16:8 method over a period of three months. Assessments were conducted at baseline, after two weeks, one month, and three months.

The study was registered in the German Clinical Trial Register (DRK S00025459) and ethical approval was obtained from the Ethics Committee of the state medical association of Rheinland-Pfalz, Germany (2020-15136).

#### 2.1.1. Participants

Adult predominantly healthy female and male volunteers were recruited for this study. Exclusion criteria were chosen to minimize the risk of serious clinical conditions as well as to exclude metabolic diseases which could influence the impact and metabolism of IF as shown in Table 1.

#### 2.1.2. Intervention

The 16:8 regimen was chosen for the IF diet, including 16 h of fasting and 8 h of allowed calorie intake on at least five days per week. Fasting days and daily fasting periods could be chosen freely by the participants to increase the practicability in everyday life and thus adherence to the diet. Once chosen, all participants were encouraged to stick to the same 16-h interval. During the 16-h fasting period, no caloric intake was allowed. Caloric drinks (e.g., soft drinks, juices), coffee additives such as sugar, syrup, or milk were prohibited. Clear broth, black coffee, and diet drinks (zero calorie drinks) were allowed during the fasting period. In the 8-h eating period, no dietary restrictions with regards to type, amount and frequency of food intake were mandated. Each participant received a thorough introduction to IF and nutritional counselling, including allowed and prohibited food or drink intake during fasting and eating periods, and was given the chance to discuss any remaining questions with a medical doctor before starting the study.

#### 2.1.3. Outcomes

**Figure 1:**
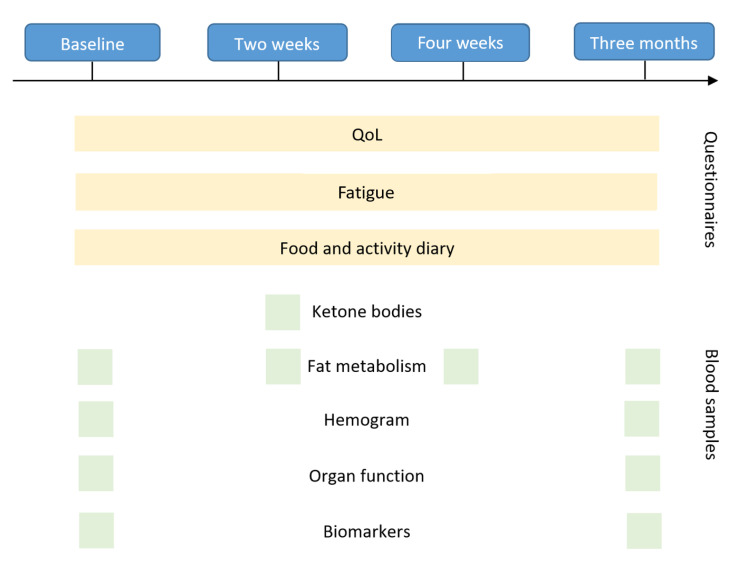
Outcome measures assessed during each study visit.

Main outcomes included short-, intermediate-, and long-term changes in overall QoL and, in particular, fatigue. Furthermore, laboratory changes with regard to the general health status, well-being, the safety of the diet, and possible factors associated with chronic diseases were assessed (Figure 1). Due to the exploratory nature of the study, some blood values were taken out of the panel based on an initial analysis of a few patients to increase cost-effectiveness.

#### 2.1.4. Quality of Life

The QoL was assessed by validated questionnaires. Because commonly used questionnaires for QoL are often validated for a specific time span, a combination of a short-term questionnaire World Health Organization Well-being index (WHO-5: two-week changes) and a long-term questionnaire Short Form Health 36 (SF-36: four weeks) were chosen. 

### 2.2. WHO-5 Well-Being Index (WHO-5)

The WHO-5 questionnaire is the most widely used outcome measure assessing subjective psychological well-being in the last two weeks [31]. The questionnaire includes five simple items related to the general mood, vitality, curiosity, and well-being balancing the wanted and unwanted effects of treatments and can be performed in less than one minute making it a good option during the clinical routine. Items are rated on a six-point scale from “All of the time” to “At no time”. Examples of the items include: I have felt active and vigorous, or I have felt calm and relaxed. The full questionnaire can be found in Supplementary Figure S1. 

### 2.3. Short Form Health 36 (SF-36)

The SF-36 questionnaire is a brief self-administrated questionnaire that covers eight dimensions of the general health status (Social Functioning, Physical Functioning, Physical Health, Mental Health, Vitality, Emotional Wellbeing, Pain, and Health Perception) in a multi-item scale with a total of 36 questions, allowing for a more detailed analysis [32]. Each item sub-score ranges from zero (heavily impaired) to 100 (no health restrictions) points. An exemplary question of this questionnaire would be: Does your health now limit you in these activities? The full questionnaire can be found in Supplementary Figure S2. The SF-36 covers a time period of the last four weeks.

#### Fatigue

Fatigue being a common factor in reduced QoL, it was evaluated with the validated questionnaires Fatigue Severity Scale (FSS) and Fatigue Assessment Scale (FAS) [33,34]. 

### 2.4. Fatigue Severity Scale (FSS)

The FSS focuses on the impact of fatigue on various aspects of life, such as work and social life as well as the ability to meet obligations [35]. We preferred multi-item scores to highly practicable single-item scores in this trial to accurately represent the complexity and different aspects of each outcome. The FSS contains nine statements that rate the severity of fatigue symptoms, with seven possible graduations (from one = disagree to seven = agree). A total score of less than 36 suggests that the respondent is not suffering from fatigue and more than 36 points suggest a significant level of fatigue. An exemplary item can be: I am easily fatigued, or Fatigue causes frequent problems for me. The full questionnaire can be found in Supplementary Figure S3, and was assessed at baseline, two weeks, and three months. 

### 2.5. Fatigue Assessment Scale (FAS)

The FAS questionnaire is a 10-item scale evaluating symptoms of chronic fatigue [34]. It includes items measuring both physical and mental fatigue and is a uni-dimensional construct, but it does not separate its measurements into these categories. The FAS questionnaire includes ten statements (e.g., I am bothered by fatigue or physically, I feel exhausted) and for each statement, the user can choose one out of five answer categories, varying from never (one point) to always (five points). The administration requires approximately two minutes. The full questionnaire can be found in Supplementary Figure S4 and was assessed at baseline, two weeks, and three months.

The FAS questionnaire includes items measuring both physical and mental fatigue. Multi-item scores were preferred to highly practicable single-item scores in this study to accurately represent the complexity and different aspects of each outcome. While the FAS questionnaire is primarily concerned with the expression of fatigue, the FSS focuses on the impact of fatigue on various aspects of life, such as work and social life as well as the ability to meet obligations [35].

#### 2.5.1. Laboratory Changes

To detect changes in metabolism, biomarkers and organ functions, peripheral blood samples were drawn.

#### 2.5.2. Adherence and Practicability

To assess behavioural changes related to food and drink choices, daily activity, sleep quality, emotional well-being, and general motivation related to the IF diet, a new questionnaire specific to this trial was designed and participants were required to keep a food and activity diary.

The Quality of Life (QoL) and fatigue questionnaires were completed once at baseline, after two weeks, after four weeks, and after three months of fasting. The questionnaires always assessed the general QoL and fatigue within the past weeks (for detailed information please refer to the description of each questionnaire in the methods section). The food and activity diary were to be completed daily by the participants. Laboratory values were assessed at baseline, two weeks, four weeks, and after three months depending on the type of blood test, as marked in the figure. 

#### 2.5.3. Statistical Analysis

The study is exploratory. With 27 participants, the study was designed to detect effect sizes of 0.5 with a power of 80% by using a one-sided *t*-test based on the results of previous Ramadan fasting trials [29,36]. The significance level was set to 5%. To account for high dropout rates with dietary interventions a total of 35 participants were included in the study [37].

The statistical tests and graphs were performed with SPSS (version 27.0.1, SPSS Inc., Chicago, IL, USA) and Stata1C (version 17 V5, Stata Corp, Texas, TX, USA). Categorical data are displayed as absolute and relative frequencies, and continuous data either with median and interquartile range or mean and standard deviation. Laboratory values, QoL, and fatigue questionnaires were compared using dependent *t*-tests. The significance level was set at 5%. Due to the exploratory character of the study, no adjustments were made for multiple testing.

## 3. Results

**Figure 2:**
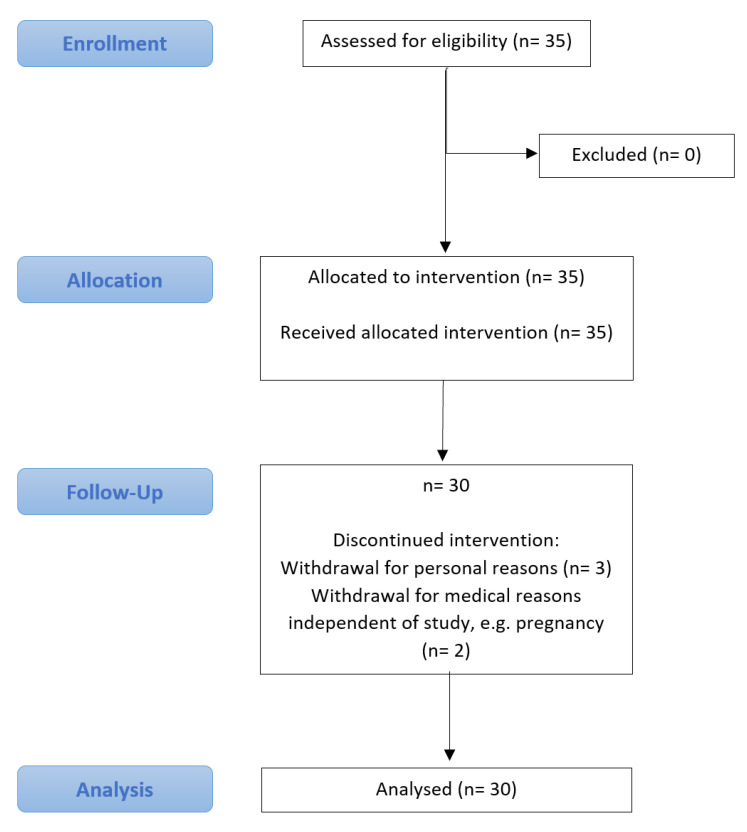
Consort flow diagram.

A total of 35 participants, including 32 female and 3 male participants between the ages of 18 to 59, were recruited for this trial. Due to a drop-out rate of 14.3% (five participants), only 30 participants were included in the analysis. A detailed CONSORT flowchart is shown in Figure 2. 

The study population was predominantly females between 20–40 years old. However, 25% were above the age of 40. The BMI ranged from 18–46.2 kg/m^2^. None of the participants suffered from Diabetes mellitus type II; however, five participants could be categorized as prediabetic with an HbA1c value of 5.7–6.2% at baseline. Interestingly, after three months of IF only two participants could still be categorized as prediabetic with HbA1c values of 5.7–5.8%. With regard to the work environment, half of the population worked for more than 40 h per week. Sedentary and active work environments were balanced in this cohort. The majority reported a varied or balanced diet and about 60% of the participants had tried a specific diet prior to this trial. Around 25% reported weight fluctuations. Baseline characteristics of all trial participants can be found in Table 2. Histograms of the baseline characteristics can be found in Supplementary Figure S5.

### 3.1. Quality of Life

**Figure 3:**
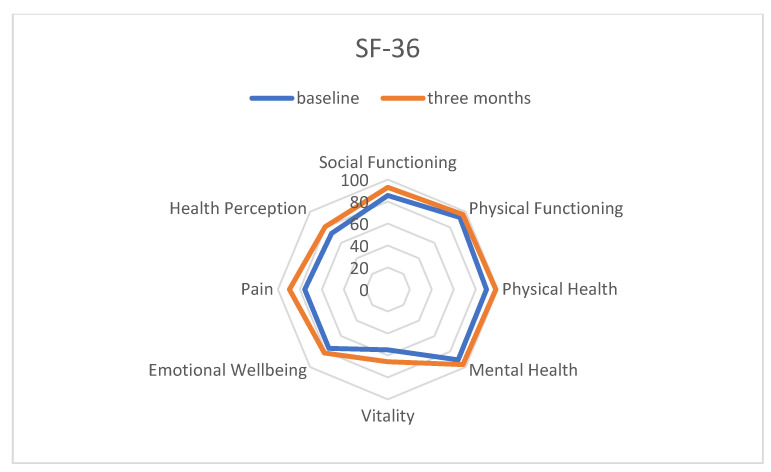
Three-month changes in Quality of Life through intermittent fasting assessed by the Short Form-36 questionnaire.

The SF-36 questionnaire contains eight categories, of which six showed significantly improved results after three months of IF e.g., n Physical Health (92.3 ± 11.6 vs. 96.5 ± 6.3; *p* = 0.015), Vitality (54.5 ± 21.2 vs. 65.8 ± 15.5, *p* < 0.001), Mental Health (75.5 ± 12.0 vs. 81.7 ± 9.0, *p* < 0.001), Social Functioning (83.9 ± 18.9 vs. 92.9 ± 9.2, *p* = 0.021), Physical Pain (74.1 ± 31.8 vs. 89.5 ± 14.9; *p* = 0.008), and General Health Perception (71.8 ± 15.5 vs. 80.4 ± 13.9, *p* = 0.001), as illustrated in Figure 3.

**Figure 4:**
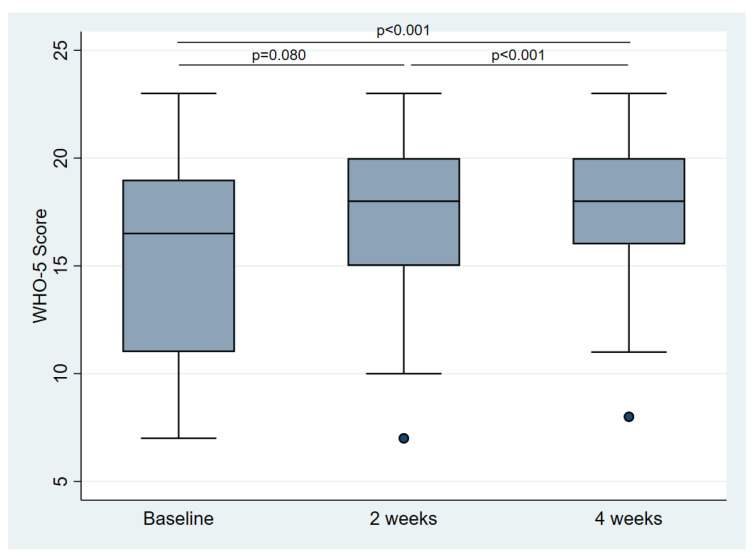
Changes in Quality of Life through intermittent fasting assessed by the WHO-5 questionnaire.

Similar results were obtained from the WHO-5 questionnaire, which registered a significant improvement in well-being comparing the baseline and four weeks (15.6 ± 4.6 vs. 18.0 ± 3.6; *p* < 0.001). However, while the positive influence of IF was already observed in many participants after two weeks, it took four weeks to develop in others. Since the WHO-5 questionnaire is only validated for short-term changes (maximum of two weeks) it was only assessed at baseline, two weeks, and four weeks. The results are shown in Figure 4. 

### 3.2. Fatigue

**Figure 5:**
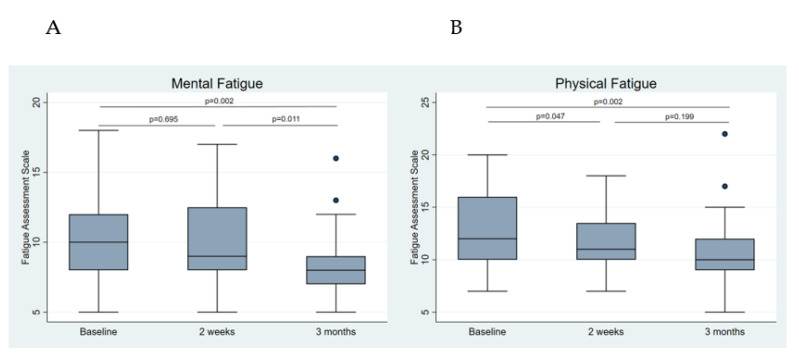
Changes in subclassifications of fatigue through intermittent fasting assessed by the Fatigue Assessment Scale (FAS). (**A**) Mental fatigue changes. (**B**) Physical fatigue changes.

The FAS questionnaire distinguishes between mental and physical fatigue (the highest score for each category is 25, which indicates more fatigue). While after two weeks no significant reduction was seen for mental fatigue, both were significantly reduced after three months of IF (mental fatigue: 10.3 ± 3.2 vs. 8.4 ± 2.5; *p* = 0.002; physical fatigue: 12.6 ± 3.8 vs. 10.7 ± 3.3; *p* = 0.002) (Figure 5). While 51.7% of the participants were classified as fatigued (of those, 20.0% were severely fatigued) at baseline, we observed a reduction to 31.0% being fatigued (of those, 11.1% were severely fatigued) after three months of IF. This amounts to a relative reduction of 40.0%.

Categorized by the FSS, 10% fell into the “significantly fatigued” category at baseline, which was reduced to 3.3% after three months of IF. This amounts to a reduction of 66.6% in severely fatigued participants through IF. The mean FSS score at baseline was 3.5 ± 1.2 (1 = minimum, 7 = maximum) and 2.9 ± 1.1 after 3 months (*p* < 0.001).

### 3.3. Safety Profile and Metabolic Changes

Overall, IF demonstrated a good safety profile. None of the participants had to terminate the trial due to side effects from IF. The Body Mass Index (BMI) was significantly reduced, but no critical or clinically relevant weight loss endangering the safety of the participants was recorded (25.5 kg/m^2^ ± 5.8 vs. 25.0 kg/m^2^ ± 5.5, *p* = 0.008). No participants with a BMI of less than 25 kg/m^2^ at the beginning of the trial lost more than 0.8 BMI-points after three months of IF and no one dropped to a BMI of below 18 kg/m^2^. None of the laboratory values reflecting organ functions (e.g., creatinine or liver enzymes) significantly worsened during this trial; by contrast, the Aspartate Aminotransferase (AST/GOT) significantly decreased. Overall, positive changes such as an increased HDL and reduced LDL were observed but failed to reach significance. The mean values of the haematological parameters (erythrocytes, haemoglobin, platelets, etc.) were within the respective reference ranges before the start of fasting and after three months of fasting with no relevant alterations. Interestingly, IGF-1 significantly reduced from 229.8 ng/mL ± 90.0 vs. 205.9 ng/mL ± 60.7 (*p* = 0.022) after three months of IF. The laboratory values can be found in Supplementary Tables S1–S3.

### 3.4. Adherence and Practicability

The practicability of the fasting concept was rated with a mean score of 7.4 on a scale from one to ten, with one being the least practical and ten being the most practical. A total of 14.3% of the subjects terminated the study prematurely. Three of five of the dropouts cited personal reasons for the discontinuation, whereas two had to leave the study due to the occurrence of health-related concerns such as pregnancy, which were independent of the study (Figure 2). According to the subjects, the extent of thoughts about food decreased significantly over the course of two weeks from the start of fasting until the end of the period of observation (5.79 ± 2.57 vs. 4.55 ± 2.38, *p* = 0.005). The feeling of hunger during the fasting period showed no difference (3.41 ± 2.47 vs. 3.28 ± 2.48, *p* = 0.786). Over 85% of the participants wanted to continue practising IF after completion of the trial, although some wanted to adhere to the schedule less strictly.

## 4. Discussion

This prospective cohort study demonstrated the positive impact of IF on QoL in a generally healthy study population. Improvement was seen over a course of three months in several aspects of QoL including physical, mental, and general health, as well as vitality and social functioning according to everyday life structures and working procedures. Fatigue was also significantly reduced with IF, without interfering with daily activities including shift work. The observation of metabolic changes and electrolytes demonstrated a good safety profile of IF. Moreover, IF was associated with a significant weight loss, especially seen in overweight people, but no critical weight loss endangering the safety of the participants has been found. In addition, significantly reduced levels of the proliferative protein IGF-1, associated with tumour development, were observed. 

The term IF is used for an eating pattern without calorie restrictions, alternating between periods of fasting and eating. A widely known and practised form of IF is the religious Ramadan fasting, as one of the five pillars of the Islamic creed, where food (and drinks) can only be consumed after sunset and before sunrise. This can result in fasting periods of around 16–18 h over 29–30 consecutive days of the 9th month of the Islamic lunar calendar [38]. Several partly contradictory studies exist examining the relationship between IF during Ramadan fasting and QoL [39,40,41,42]. Participants showed disturbances in their sleep architecture, although their cognitive and physical performance was not negatively impacted during the one-month fasting period. Some trials have reported significant improvements in fatigue, measured by the visual analogue scale and the FSS as well as an elevated mood, using Beck’s Depression Index [42]. Similar to Ramadan fasting, the IF scheme used in this trial consisted of 16 h fasting periods followed by eating phases of 8 h without any caloric restrictions. However, in contrast to the fasting during Ramadan, all participants in our study chose to include the sleeping period in their fasting hours while eating throughout the day. Nevertheless, the rhythms varied. While some preferred to eat breakfast and skip dinner, others forwent breakfast. Due to the flexibility of IF, every participant was able to adjust the time periods according to his/her own preferences and daily activities. This could explain why we observed an increase in the QoL in the present trial, while many studies assessing Ramadan fasting failed to do so [43].

The main advantage of our trial compared to the existing evidence on the effect of IF on QoL, is the relatively long trial period of three months. Due to its nature, trials assessing religious fasting only observed the changes over the course of one month of fasting [1,4,40]. However, adjusting to a dietary change and successfully adapting one’s body to this new lifestyle needs time [44]. Short-term changes can result in a decreased metabolism and impaired physical and psychological status before showing their benefits [45]. In our study, we observed that the general QoL measured by the WHO-5 questionnaire was not significantly improved after two weeks, but a significant and clinically relevant increase in the QoL was seen after four weeks. Similarly, the advantages of decreasing fatigue were mostly demonstrated in the last two months of the intervention, in particular after a time of habituation and adaption of the circadian rhythm. This hypothesis is underlined by several short-term IF trials, which found no relevant changes in cognitive function, fatigue, or QoL. Nugraha et al. examined a similar fasting regimen in 50 young men fasting during a long period of 17–18 h per day during Ramadan (one month), compared to a non-fasting group [42]. They found no significant differences between the two groups with regard to mood-related symptoms, fatigue, and QoL after one month. A small study conducted by Zajac et al. designed a randomized cross-over trial with a total of 17 females, who practiced either true fasting (nil-caloric intake), modified fasting defined as bulking (two meals eaten early in the day; 512 kcal total), or modified fasting with calorie-restricted food intake on a single day of the week [25]. No differences with regard to cognitive function were found between the different fasting regimens. Subjectively, hunger was significantly higher in the true fasting group compared to both modified-fasting groups. Overall, the study groups had decreased blood glucose levels and increased hunger, whereas fatigue was only significantly increased at one point in time out of seven over a 7.5 h recorded period. Nevertheless, the results are limited by their very short fasting period of a single day with a one-week wash-out period between fasting conditions. Similar to these results, Solianik et al., who examined a two-day total fasting routine [46] showed that mood, brain activity, and cognitive as well as psychomotor performance were not affected by fasting.

By contrast, few trials assessed prolonged IF periods comparable to our three-month active trial period. A secondary analysis of two pilot studies by Kesztyüs et al. observed a similar timeline and fasting regimen over three months [47]. They included a heterogeneous study population with generally healthy employees (1st cohort) and abdominal obese patients (2nd cohort) from a general practitioner’s office. In line with our results, they demonstrated an increased QoL and increased sleep quality independent of the weight loss in both groups. However, QoL was only assessed through a visual analogue scale and detailed information on the different aspects of QoL including fatigue was not assessed, as the focus was on the quality of sleep. Teong et al. presented increased performances in cognition after IF over eight weeks. This randomized controlled trial included 46 overweight or obese women) (mean BMI 32.9 (±4.4 kg/m^2^)), of whom 23 performed IF and 23 had a calorie-restricted diet [45].

While prolonged, strict fasting periods can be very strenuous for the body and thus cannot be recommended for everyone trying to improve QoL [48], IF in the 16:8 h regimen has demonstrated a very good safety profile in this trial. In order to verify potential changes in organ functioning besides the metabolic switch, we measured some laboratory parameters such as electrolytes, functional parameters of the liver and kidneys, as well as the hemogram, and metabolic parameters (e.g., lactate, insulin levels) at multiple time points throughout the trial, which demonstrated no significant differences over time (see Supplementary Tables S1–S3). Two weeks after the start of fasting, we assessed ketone bodies to verify the metabolic switch (Supplementary Table S3). Ketone bodies are the main source of energy for maintaining bodily functions in the starvation state [4,49]. As expected, ketone bodies increased sharply from 12 to 18 h after the last caloric intake, confirming the change in metabolism. In the starvation state, ketone bodies are synthesised from acetyl-CoA in the liver, which in turn is a breakdown product of fatty acids. This metabolic process can explain the weight-reducing effect of time-restricted eating [50,51,52]. IF is not necessarily performed for weight loss, especially as no caloric restrictions were imposed in the eight hours of non-fasting. Particularly the non-obese patient population in our trial did not lose a significant amount of weight, making this form of IF safe for people even on the lower scale of a normal BMI. Similarly, Kesztyüs et al. found a greater reduction in weight in their obese population, while the mixed, average weight population experienced a reduction of only −0.4 points in BMI after three months of IF [47]. 

Fatigue and physical weakness are not only strenuous side effects of shift-work [53,54], but are also common in patients with cancer, especially when undergoing cytotoxic therapy (chemotherapy) [55,56,57]. A “healthy” diet is often thought to support the treatment process. However, at present, very little is known about the effects of different diets during chemotherapy. In this trial, we were able to demonstrate an improvement in mental and physical fatigue, and the QoL, even in a generally healthy study population. This finding raises the question of whether chemotherapy-related fatigue and QoL could be positively influenced by IF following the 16:8 method. In a few studies IF has been shown to reduce side effects in patients treated with chemotherapy [3,22,23]. Short-term continuous fasting periods of 48 h to 96 h during chemotherapy demonstrated positive effects on fatigue and QoL in the current literature [24,25,26]. Nonetheless, those trials did not assess a continuous diet modification (lifestyle change), but rather short and strict fasting interventions. Bauersfeld et al. studied the effects of short-term fasting 36 h before and 24 h after chemotherapy on QoL and tolerance of chemotherapy in a randomized cross-over pilot trial [24]. They included 34 women with gynaecological malignancies and demonstrated that short-time fasting was well tolerated and appeared to improve QoL as well as fatigue. Overall, evidence of the effects of IF on chemotherapy fatigue and QoL is very scarce.

Besides the subjectively improved QoL, we were also able to demonstrate significantly decreased levels of IGF-1. This falls in line with the results of previous trials, describing changes in IGF-1 after about 18 h of fasting [24,58]. IGF-1 plays a critical role in tumour development and progression. By lowering the activation of the mTOR signalling pathway through reduced IGF-1 levels, more proteins are reused instead of being synthesized (increased autophagy). Thus, healthy cells become more resistant to oxidative stress. Nevertheless, this self-regulating mechanism seems to be absent in tumour cells. Consequently, healthy cells may gain a survival advantage due to their increased protection and the accelerated repair of their DNA, by lowering IGF levels, for example through IF. Thus, healthy cells could be selectively protected during chemotherapy [4,10,27] and given the overall positive correlation between lower IGF-1 levels and decreased cell division activity [23,24,58] the potential of IF for patients suffering from cancer seems enormous. Overall, the 16:8 h method as practised in our trial seems especially promising for cancer patients due to its practicability and safety. However, its full effects and benefits for cancer patients need to be assessed in a larger clinical trial. 

When interpreting the results of the trial, some limitations need to be considered. To experience the full benefits of any diet modification, continuance and compliance are important. A few of the study participants reported that they were only able to follow the proposed 16:8 IF schedule on three out of seven days per week. Given the moderate size of the study population, this incompliance might have overshadowed the true benefits of IF in some respects. Strict rules and restrictions are often impracticable, especially for people with flexible working hours (e.g., shift work) and could have led to a discontinuation of a new diet; however, the 16:8 h IF schedule on five days per week showed to be feasible for the majority of our trial population. We limited the number of obligatory fasting days to five per week in order to improve compliance and practicability in daily life. On average, subjects fasted 5.5 days a week and rated the fasting regimen used at 7.4 out of 10 for feasibility after three months, with a significant increase in the rating after two weeks. The most obvious limitation of this trial results from the single-group study design. Due to the lack of a control group, no definite conclusion on the reduction of fatigue and improvement of QoL can be drawn for the general population, and larger controlled trials are needed to confirm our results. The voluntary participation in this trial with the expectation of a major change in one’s lifestyle might have influenced the results of an improved fatigue and QoL. However, no long-term dietary changes can be forced upon an individual, and anyone starting a dietary modification will do so with the goal of becoming healthier, losing weight, or increasing their well-being. Thus, a similar psychological bias could be expected from anyone in the general population. Furthermore, our study population consisted of a large proportion of women, which raises the question of whether it is representative of the average population. Due to the recruitment strategy in this trial, the examined study population was predominantly female, representing the gender ratio often found in social professions, such as nursing or social caregivers [58]. Hence, the gender distribution reflects the population in many health care professions. Furthermore, the current evidence on the effects of IF on QoL is dominated by male study populations (athletes, Ramadan fasting) [40,41], which highlights the added value of our trial to the current evidence on IF. Further prospective, randomized controlled trials are necessary to examine the influence of IF on different study populations and to better understand the mechanism behind the improved QoL through IF. It would be of great interest to directly compare various IF schedules in clinical trials in the future to identify the most beneficial and feasible fasting method to improve QoL. Due to the large number of dietary and fasting options available, these clinical trials can be the basis for ultimately finding an individualized diet approach for patients, aiming to increase not only the feasibility but possibly also the efficacy of a dietary intervention to improve one’s QoL by taking each individual’s lifestyle, diseases, and metabolic system into account. 

## 5. Conclusions

Our study confirmed that IF according to the moderate 16:8 h regimen leads to improvements in the QoL, especially physical and mental fatigue, while maintaining a good safety profile. IF has proven to be feasible in this trial, even for shift workers and people with a high percentage of manual/active labour. Thus, IF can be considered a very beneficial diet for people suffering from fatigue in stressful jobs. Furthermore, the promising results highlight the potential of IF for patients suffering from fatigue and QoL impairment due to other chronic diseases or circumstances such as chemotherapy. The demonstrated significant reduction in the proliferative marker IGF-1, vital to tumour development and progression, may be hypothesis-generating for additional benefits of IF for cancer patients.

## Figures and Tables

**Table 1:** Inclusion and exclusion criteria of the study.

Inclusion Criteria	≥18 Years of Age
	Written informed consent
	Ability to understand nature and effects of the trial participation
**Exclusion Criteria**	Body mass index <18 kg/m^2^
	Weight loss of >10% of body weight in the last 6 months
	Current or recent diet aimed at reducing weight
	Current or past eating disorders
	Acute medical conditions (including ulcus/gastritis)
	Chronic or acute heart, liver, or kidney disease
	Diabetes mellitus type I
	Large surgical procedures in the last 6 months
	Pregnancy and breastfeeding period
	Participation in another interventional trial likely to interfere with the study outcome

**Table 2:** Subjects’ characteristics (*n* = 30).

	Frequency (*n*) Percentage (%)		Frequency (*n*) Percentage (%)
**Gender** Female Male	27 (90.0%) 3 (10.0%)	**Eating habits** None in particular Vegetarian Vegan Organic products only	21 (70.0%) 5 (16.7%) 3 (10.0%) 1 (3.3%)
**Age [years]** <20 20–29 30–39 40–49 50–59	1 (3.3%) 17 (56.7%) 4 (13.3%) 4 (13.3%) 4 (13.3%)	**Meal preparation** Rarely self-prepared Often homemade Mostly home cooked Always self-prepared	0 (0.0%) 10 (33.3%) 20 (66.7%) 0 (0.0%)
**Working hours [hours/week]** <40 40–50 >50	14 (46.7%) 13 (43.3%) 3 (10.0%)	**Consumption of ready-made products** Never Rarely Often	9 (30.0%) 20 (66.7%) 1 (3.3%)
**Movement at work** Predominantly sedentary Relatively plenty of exercise	17 (56.7%) 13 (43.3%)	**Physical activity [hours/week]** <0.5 0.5 1–2 >2	6 (20.7%) 2 (6.9%) 6 (20.7%) 15 (51.7%)
**Nutritional awareness** Yes No	17 (60.7%) 11 (39.3%)	**Weight fluctuations** yes no	4 (13.3%) 26 (86.7%)
**Diet** Rather one-sided Varies Balanced	1 (3.3%) 16 (53.3%) 13 (43.3%)	**First diet** yes no	11 (40.7%) 16 (59.3%)

## Data Availability

The data presented in this study are available on request from the corresponding author.

## Supplementary Materials

The following supporting information can be downloaded at: https://www.mdpi.com/article/10.3390/nu14194216/s1, Table S1: Laboratory values: General; Table S2: Laboratory values: Fat metabolism; Table S3: Laboratory values: Metabolic parameters; Figure S1: World Health Organization Well-being Index (WHO-5); Figure S2: Short Form Health 36 (SF-36); Figure S3: Fatigue Severity Scale (FSS); Figure S4: Fatigue Assessment Scale (FAS); Figure S5: Baseline characteristics: Histograms.
