# Supplementary material for: Intermittent Fasting—Short- and Long-Term Quality of Life, Fatigue, and Safety in Healthy Volunteers: A Prospective, Clinical Trial
Source: Nutrients. 2022 Oct 10;14(19):4216. doi: 10.3390/nu14194216 (PMC9571750; doi:10.3390/nu14194216)

Supplementary material

Anic, K.; Schmidt, M.W.; Furtado, L.; Weidenbach, L.; Battista, M.J.; Schmidt, M.; Schwab, R.; Brenner, W.; Ruckes, C.; Lotz, J.; et al. Intermittent fasting – Short- and long-term quality of life, fatigue and safety in healthy volunteers: a prospective, clinical trial.

Table of contents:

Table S1: Laboratory values: General

Table S2: Laboratory values: Fat metabolism

Table S3: Laboratory values: Metabolic parameters

Figure S1: World Health Organization Well-being Index (WHO-5)

Figure S2: Short Form Health 36 (SF-36)

Figure S3: Fatigue Severity Scale (FSS)

Figure S4: Fatigue Assessment Scale (FAS)

Figure S5: Baseline characteristics: Histograms

Table S1: Laboratory values: General

	Baseline T0		T3		p- value T0-T3
	Mean	SD	Mean	SD	
**Electrolytes**					
Natrium	139.25	1.75	139.88	1.81	0.370
Kalium	4.16	0.29	4.04	0.16	0.421
Calcium	2.33	0.08	2.41	0.07	0.029
Magnesium	0.81	0.06	0.84	0.05	0.380
Chlorid	105.03	2.11	103.75	1.58	0.394
**Organ functions**					
Creatinine	-2.61	18.21	N/A	N/A	N/A
Uric acid	4.46	1.02	N/A	N/A	N/A
ALAT	24.4	19.74	19.48	8.57	0.179
ASAT	28.53	14.41	23.84	4.93	0.177
Bilirubin	0.74	0.49	0.77	0.46	0.809
**Hemogram**					
Leukocysts	6.23	1.69	6.36	1.55	0.736
Erythrocytes	4.62	0.44	4.64	0.37	0.695
Hemoglobin	13.46	1.46	13.45	1.40	0.946
Hematocrit	39.96	3.83	40.04	3.49	0.873
Thrombocytes	260.03	53.86	264.93	54.82	0.565
**Biomarkers**					
CRP	4.49	7.96	2.22	2.98	0.137
HbA1c	32.54	3.74	32.5	3.02	0.934
IGF-1	229.8	90.0	205.9	60.7	0.022

Table S2: Laboratory values: Fat metabolism

	**Baseline T0**		**T1**		**T2**		**T3**		**p- value T0-T3**
	Mean	SD	Mean	SD	Mean	SD	Mean	SD	
Triglycerid	92.2	40.2	91.1	51.9	97	39.6	104.6	61.41	0.510
Total cholesterol	201.7	40.1	184.3	67.8	195.8	39.9	195.4	47.67	0.462
HDL	64.9	13.9	65.5	48.7	62.4	13.8	72.72	42.58	0.254
LDL	118.4	32.0	104.2	46.4	114	32.5	112.36	30.64	0.453
HDL/LDL	1.9	0.6	1.9	0.6	1.9	0.6	1.88	0.57	0.944

Table S3: Laboratory values: metabolic parameters (assessed at four weeks)

Fasting time	12h		14h		16h		18h	
	Mean	SD	Mean	SD	Mean	SD	Mean	SD
Lactat	1.0	0.4	1.0	0.6	1.5	0.7	1.04	1.1
Cortisol	16.0	5.6	10.6	3.9	10.1	3.7	8.8	3.3
Insuline	5.5	2.1	5.4	1.9	4.7	1.1	N/A	N/A
Ketone	70.5	109.5	150.2	180.0			277	269.9

Figure S1: World Health Organization Well-being Index (WHO-5)

WHO-5 Well-being Index

**Please respond to each item by marking <u>one box per row</u>, regarding how you felt in the last two weeks.**		**All of the time**	**Most of the time**	**More than half the time**	**Less than half the time**	**Some of the time**	**At no time**
WHO 1	I have felt cheerful in good spirits.	☐ 5	☐ 4	☐ 3	☐ 2	☐ 1	☐ 0
WHO 2	I have felt calm and relaxed.	☐ 5	☐ 4	☐ 3	☐ 2	☐ 1	☐ 0
WHO 3	I have felt active and vigorous.	☐ 5	☐ 4	☐ 3	☐ 2	☐ 1	☐ 0
WHO 4	I woke up feeling fresh and rested.	☐ 5	☐ 4	☐ 3	☐ 2	☐ 1	☐ 0
WHO 5	My daily life has been filled with things that interest me.	☐ 5	☐ 4	☐ 3	☐ 2	☐ 1	☐ 0

Scoring:
The raw score is calculated by totaling the figures of the five answers. The raw score ranges from 0 to 25, 0 representing worst possible and 25 representing best possible quality of life.

To obtain a percentage score ranging from 0 to 100, the raw score is multiplied by 4. A percentage score of 0 represents worst possible, whereas a score of 100 represents best possible quality of life.

Regional Office for Europe WHO. Use of Well-Being Measures in Primary Health Care - The DepCare Project. Health for All, Target 12, 1998 [http://www.who.dk/document/e60246.pdf]

Bech P. Measuring the dimensions of psychological general well-being by the WHO-5. QoL Newsletter 2004; 32: 15-16.

Figure S2: Short Form Health 36 (SF-36)

Please answer the 36 questions of the **Health Survey** completely, honestly, and without interruptions.

GENERAL HEALTH:

In general, would you say your health is:

◯ Excellent ◯ Very Good ◯ Good ◯ Fair ◯ Poor

Compared to one year ago, how would you rate your health in general now?

◯ Much better now than one year ago
◯ Somewhat better now than one year ago
◯ About the same
◯ Somewhat worse now than one year ago
◯ Much worse than one year ago

LIMITATIONS OF ACTIVITIES:

The following items are about activities you might do during a typical day. Does your health now limit you in these activities? If so, how much?

Vigorous activities, such as running, lifting heavy objects, participating in strenuous sports.

◯ Yes, Limited a lot ◯ Yes, Limited a Little ◯ No, Not Limited at all

Moderate activities, such as moving a table, pushing a vacuum cleaner, bowling, or playing golf

◯ Yes, Limited a Lot ◯ Yes, Limited a Little ◯ No, Not Limited at all

Lifting or carrying groceries

◯ Yes, Limited a Lot ◯ Yes, Limited a Little ◯ No, Not Limited at all

Climbing several flights of stairs

◯ Yes, Limited a Lot ◯ Yes, Limited a Little ◯ No, Not Limited at all

Climbing one flight of stairs

◯ Yes, Limited a Lot ◯ Yes, Limited a Little ◯ No, Not Limited at all

Bending, kneeling, or stooping

◯ Yes, Limited a Lot ◯ Yes, Limited a Little ◯ No, Not Limited at all

Walking more than a mile

◯ Yes, Limited a Lot ◯ Yes, Limited a Little ◯ No, Not Limited at all

Walking several blocks

◯ Yes, Limited a Lot ◯ Yes, Limited a Little ◯ No, Not Limited at all

Walking one block

◯ Yes, Limited a Lot ◯ Yes, Limited a Little ◯ No, Not Limited at all

Bathing or dressing yourself
◯Yes, Limited a Lot ◯Yes, Limited a Little ◯No, Not Limited at all

PHYSICAL HEALTH PROBLEMS:
During the past 4 weeks, have you had any of the following problems with your work or other regular daily activities as a result of your physical health?

Cut down the amount of time you spent on work or other activities
◯Yes ◯No

Accomplished less than you would like
◯Yes ◯No

Were limited in the kind of work or other activities
◯Yes ◯No

Had difficulty performing the work or other activities (for example, it took extra effort)
◯Yes ◯No

EMOTIONAL HEALTH PROBLEMS:
During the past 4 weeks, have you had any of the following problems with your work or other regular daily activities as a result of any emotional problems (such as feeling depressed or anxious)?

Cut down the amount of time you spent on work or other activities
◯Yes ◯No

Accomplished less than you would like
◯Yes ◯No

Didn't do work or other activities as carefully as usual
◯Yes ◯No

SOCIAL ACTIVITIES:
Emotional problems interfered with your normal social activities with family, friends, neighbors, or groups?

◯Not at all ◯Slightly ◯Moderately ◯Severe ◯Very Severe

PAIN:
How much bodily pain have you had during the past 4 weeks?

◯None ◯Very Mild ◯Mild ◯Moderate ◯Severe ◯Very Severe

During the past 4 weeks, how much did pain interfere with your normal work (including both work outside the home and housework)?

◯Not at all ◯A little bit ◯Moderately ◯Quite a bit ◯Extremely

ENERGY AND EMOTIONS:
These questions are about how you feel and how things have been with you during the last 4 weeks. For each question, please give the answer that comes closest to the way you have been feeling.

Did you feel full of pep?
- All of the time
- Most of the time
- A good Bit of the Time
- Some of the time
- A little bit of the time
- None of the Time

Have you been a very nervous person?
- All of the time
- Most of the time
- A good Bit of the Time
- Some of the time
- A little bit of the time
- None of the Time

Have you felt so down in the dumps that nothing could cheer you up?
- All of the time
- Most of the time
- A good Bit of the Time
- Some of the time
- A little bit of the time
- None of the Time

Have you felt calm and peaceful?
- All of the time
- Most of the time
- A good Bit of the Time
- Some of the time
- A little bit of the time
- None of the Time

Did you have a lot of energy?
- All of the time
- Most of the time
- A good Bit of the Time
- Some of the time
- A little bit of the time
- None of the Time

Have you felt downhearted and blue?

- ◯ All of the time
- ◯ Most of the time
- ◯ A good Bit of the Time
- ◯ Some of the time
- ◯ A little bit of the time
- ◯ None of the Time

Did you feel worn out?

- ◯ All of the time
- ◯ Most of the time
- ◯ A good Bit of the Time
- ◯ Some of the time
- ◯ A little bit of the time
- ◯ None of the Time

Have you been a happy person?

- ◯ All of the time
- ◯ Most of the time
- ◯ A good Bit of the Time
- ◯ Some of the time
- ◯ A little bit of the time
- ◯ None of the Time

Did you feel tired?

- ◯ All of the time
- ◯ Most of the time
- ◯ A good Bit of the Time
- ◯ Some of the time
- ◯ A little bit of the time
- ◯ None of the Time

SOCIAL ACTIVITIES:
During the past 4 weeks, how much of the time has your physical health or emotional problems interfered with your social activities (like visiting with friends, relatives, etc.)?

- ◯ All of the time
- ◯ Most of the time
- ◯ Some of the time
- ◯ A little bit of the time
- ◯ None of the Time

GENERAL HEALTH:
How true or false is each of the following statements for you?

I seem to get sick a little easier than other people
○ Definitely true ○ Mostly true ○ Don't know ○ Mostly false ○ Definitely false

I am as healthy as anybody I know
○ Definitely true ○ Mostly true ○ Don't know ○ Mostly false ○ Definitely false

I expect my health to get worse
○ Definitely true ○ Mostly true ○ Don't know ○ Mostly false ○ Definitely false

My health is excellent
○ Definitely true ○ Mostly true ○ Don't know ○ Mostly false ○ Definitely false

Ware Jr, J. E. (2000). SF-36 health survey update. *Spine*, *25*(24), 3130-3139.

Figure S3: Fatigue Severity Scale (FSS)

Fatigue Severity Scale (FSS, English version)*

	strongly disagree 1	2	3	4	5	6	***strongly agree*** 7
1. My motivation is lower when I am fatigued.	0	0	0	0	0	0	0
2. Exercise brings on my fatigue.	0	0	0	0	0	0	0
3. I am easily fatigued.	0	0	0	0	0	0	0
4. Fatigue interferes with my physical functioning.	0	0	0	0	0	0	0
5. Fatigue causes frequent problems for me.	0	0	0	0	0	0	0
6. My fatigue prevents sustained physical functioning.	0	0	0	0	0	0	0
7. Fatigue interferes with carrying out certain duties and responsibilities.	0	0	0	0	0	0	0
8. Fatigue is among my three most disabling symptoms.	0	0	0	0	0	0	0
9. Fatigue interferes with my work, family, or social life.	0	0	0	0	0	0	0

**Patients are instructed to choose a number from 1 to 7 that indicates their degree of agreement with each statement where 1 indicates strongly disagree and 7, strongly agree.* [Krupp et al, Arch Neurol 1989]

Figure S4: Fatigue Assessment Scale (FAS)

Fatigue Assessment Scale (FAS)

The following ten statements refer to how you usually feel. Per statement you can choose one out of five answer categories, varying from Never to Always.
Please circle the answer to each question that is applicable to you. Please give an answer to each question, even if you do not have any complaints at the moment.

1. **Never**
2. **Sometimes** (about monthly or less)
3. **Regularly** (about a few times a month)
4. **Often** (about weekly)
5. **Always** (about every day)

		Never	Sometimes	Regularly	Often	Always
1.	I am bothered by fatigue	○	○	○	○	○
2.	I get tired very quickly	○	○	○	○	○
3.	I don't do much during the day	○	○	○	○	○
4.	I have enough energy for everyday life	○	○	○	○	○
5.	Physically, I feel exhausted	○	○	○	○	○
6.	I have problems to start things	○	○	○	○	○
7.	I have problems to think clearly	○	○	○	○	○
8.	I feel no desire to do anything	○	○	○	○	○
9.	Mentally, I feel exhausted	○	○	○	○	○
10.	When I am doing something, I can concentrate quite well	○	○	○	○	○

References

1. Drent M, Lower EE, De Vries J. Sarcoidosis-associated fatigue. Eur Respir J 2012; 40: 255–263. http://www.ncbi.nlm.nih.gov/pubmed/22441750
2. Kleijn WPE, De Vries J, Wijnen PAHM, Drent M. Minimal (clinically) important differences for the Fatigue Assessment Scale in sarcoidosis. Respir Med 2011; 105: 1388-95. http://www.ncbi.nlm.nih.gov/pubmed/21700440
3. De Vries, Michielsen H, Van Heck GL, Drent M. Measuring fatigue in sarcoidosis: the Fatigue Assessment Scale (FAS). Br J Health Psychol 2004; 9: 279-91. http://www.ncbi.nlm.nih.gov/pubmed/15296678
4. Hendriks C, Drent M, Elfferich M, De Vries J. The Fatigue Assessment Scale (FAS): quality and availability in sarcoidosis and other diseases. Curr Opin Pulm Med 2018; 24 (5): 495-503. https://pubmed.ncbi.nlm.nih.gov/29889115

ild care foundation

© ild care foundation; www.ildcare.nl; usage is only allowed with the permission of the ild care foundation: info@ildcare.nl.

Figure S5: Baseline characteristics: Histograms

Gender

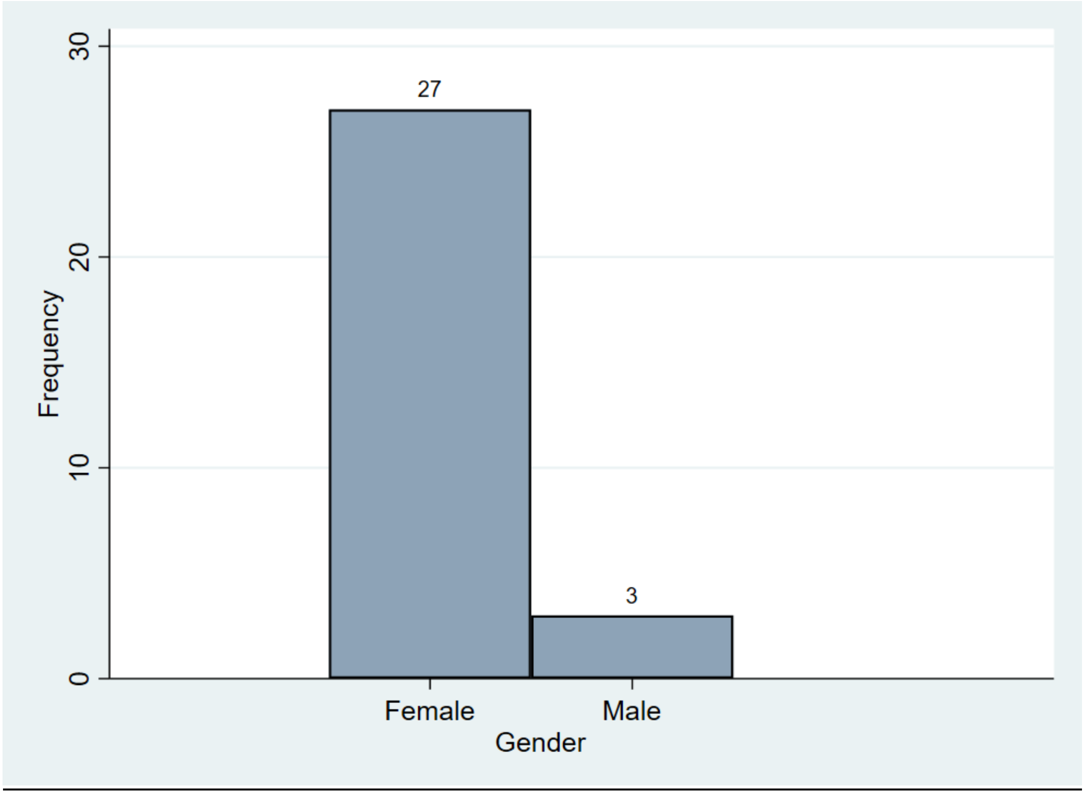

Age

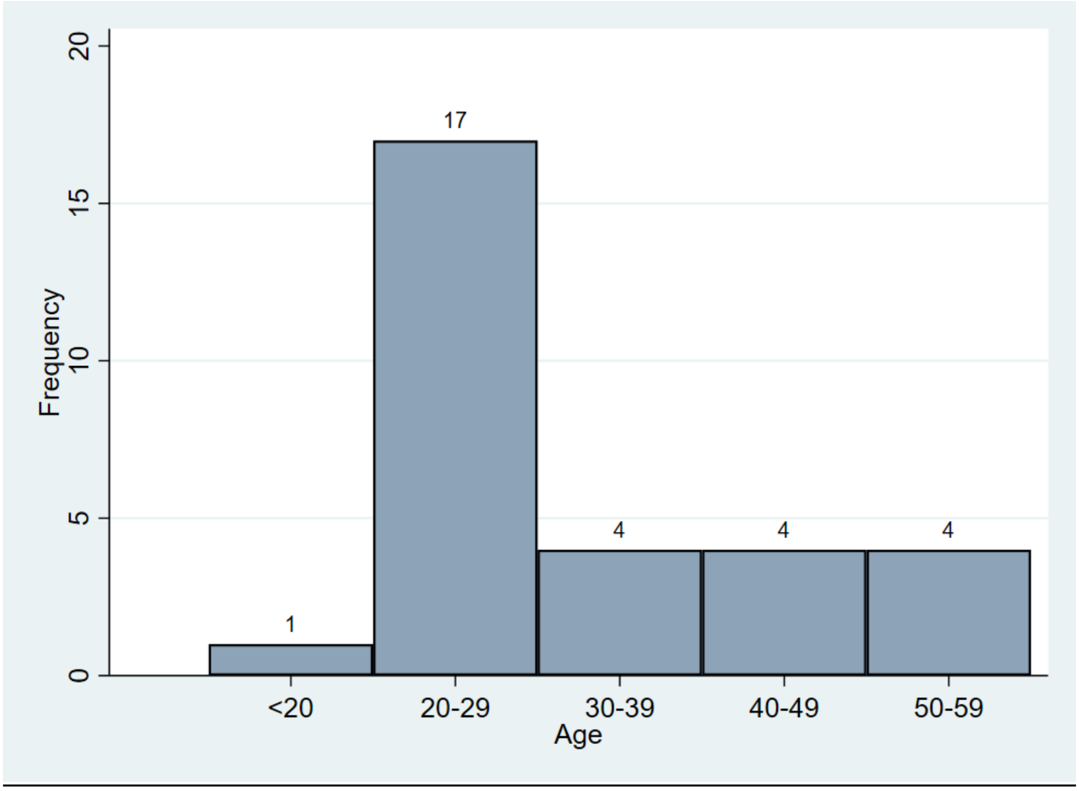

Working hours

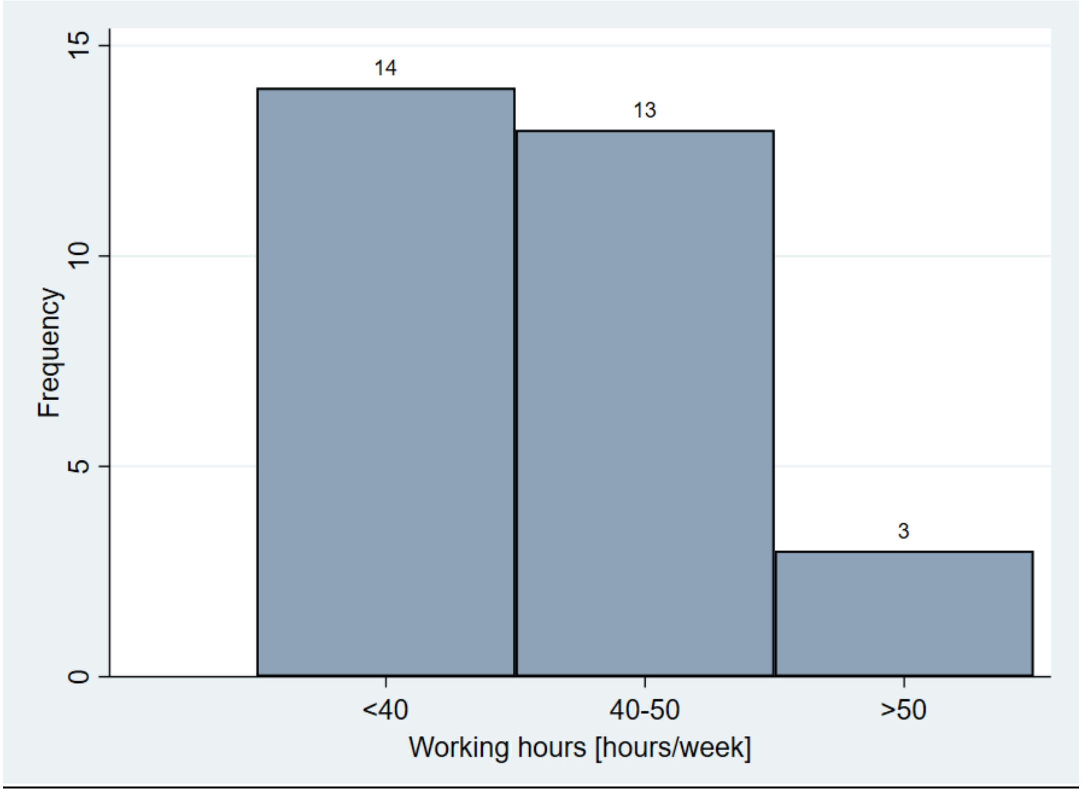

Movement at work

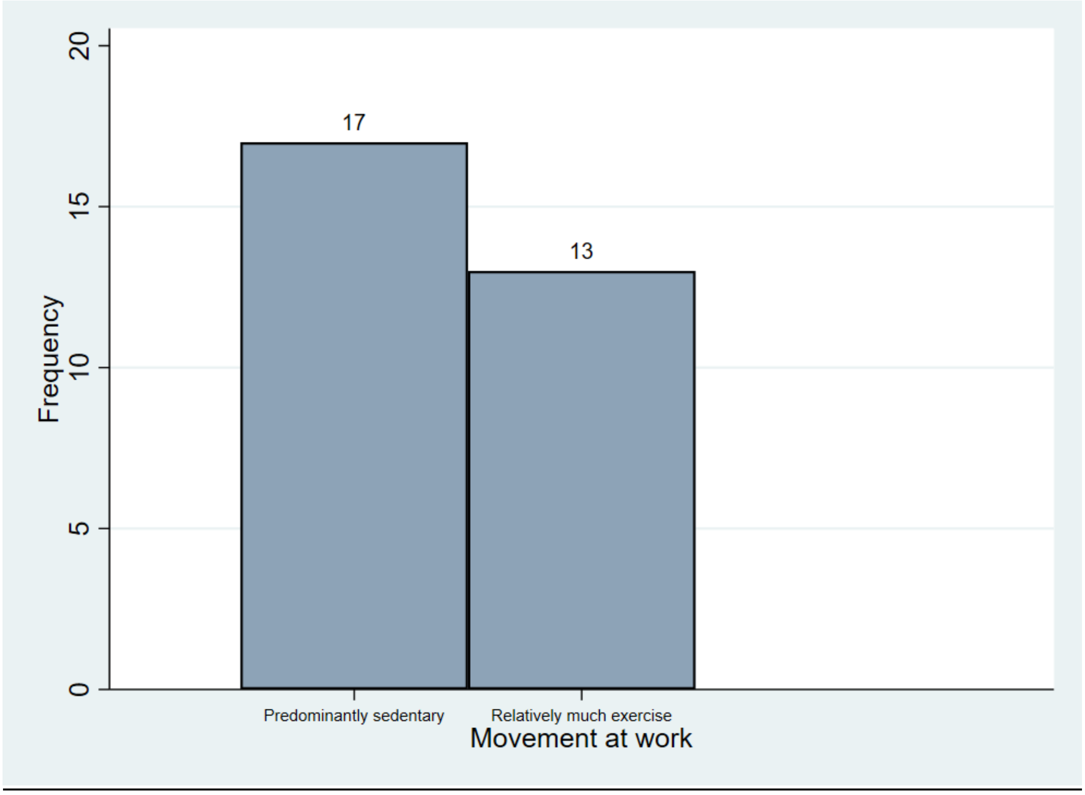

Nutritional awareness

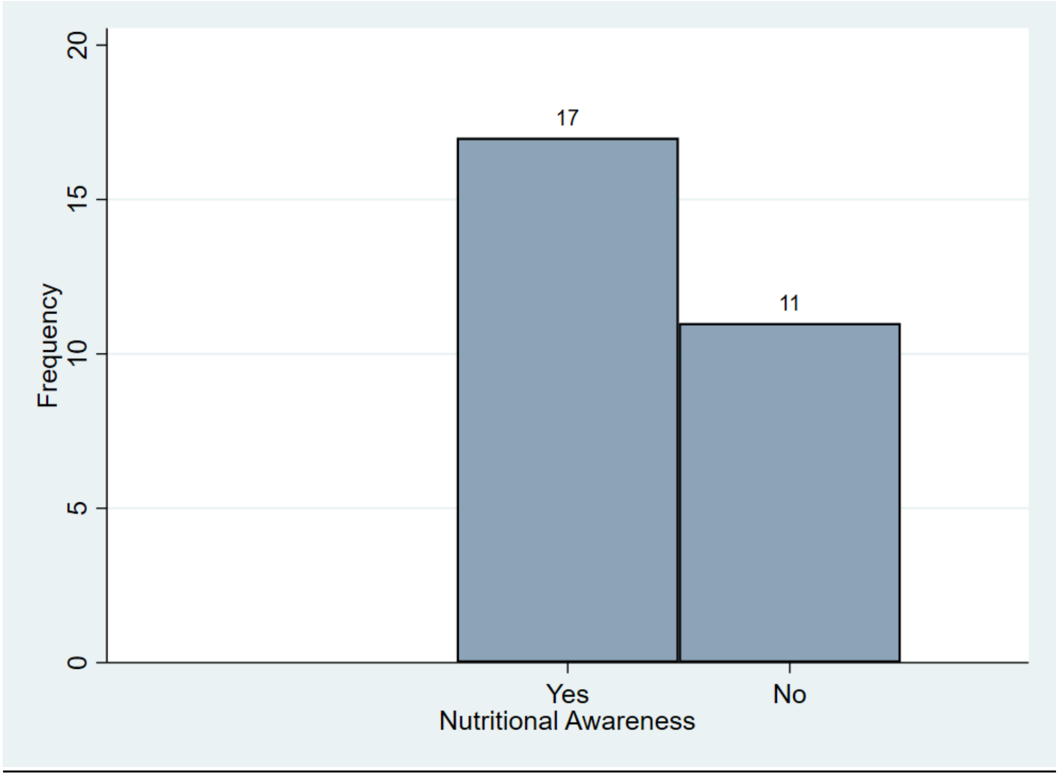

Eating habits

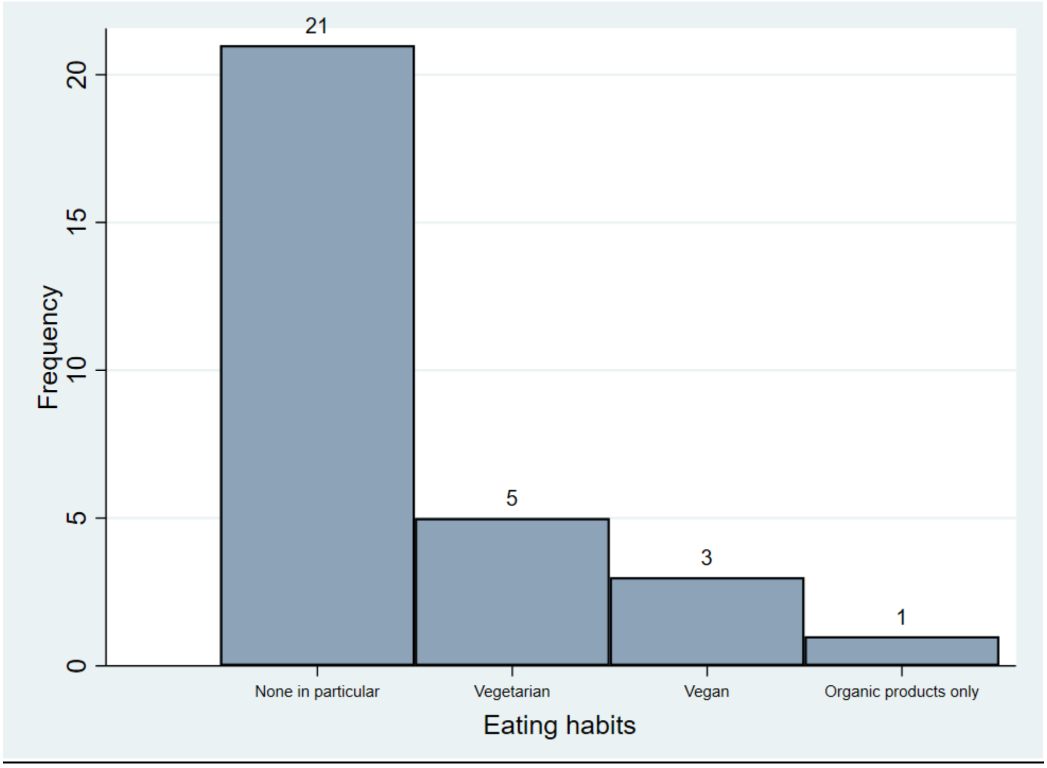

Meal preparation

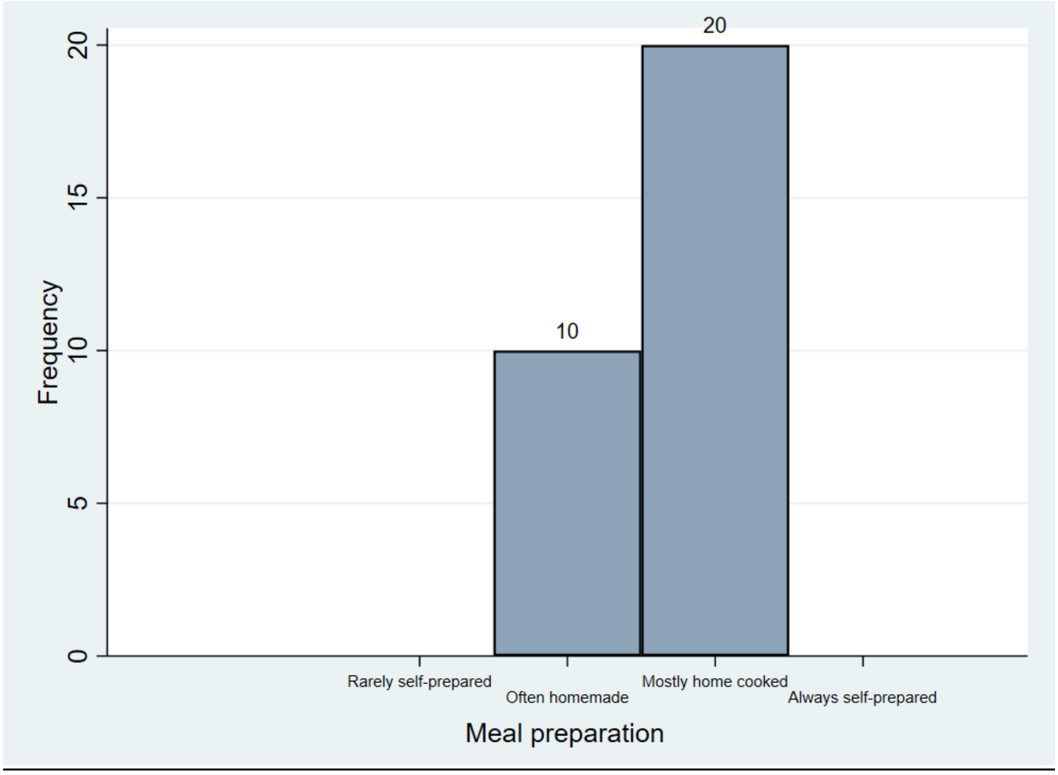

Consumption of ready-made products

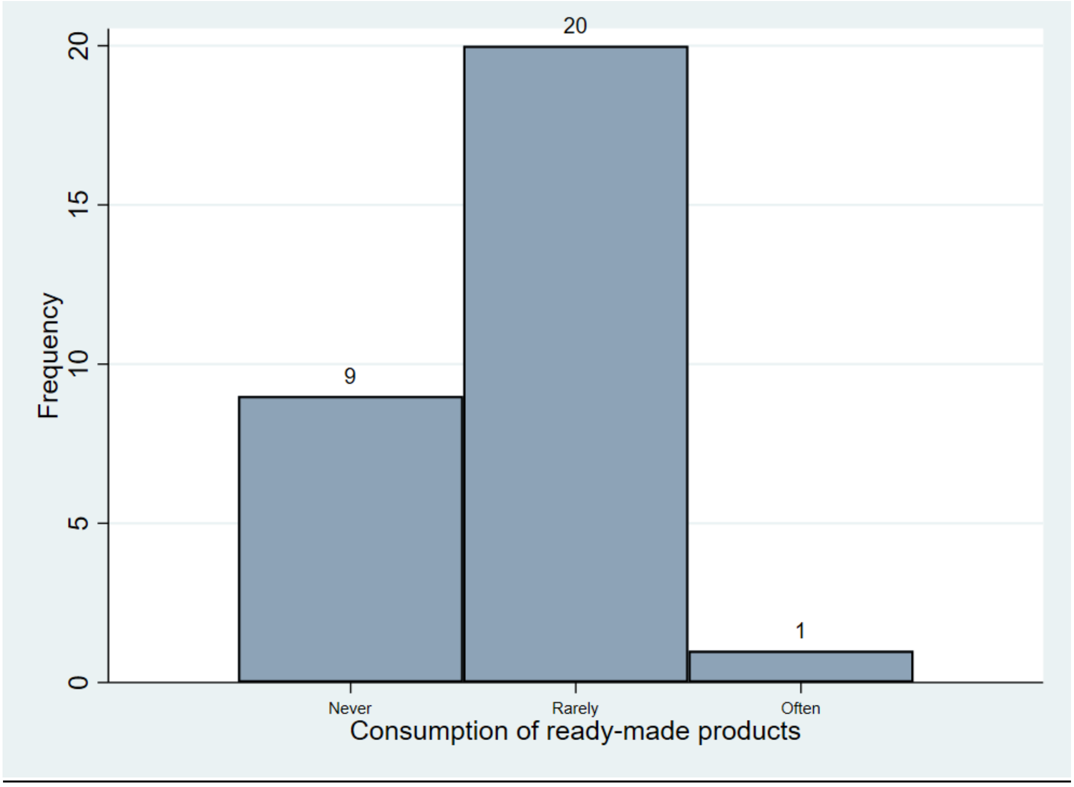

Physical activity

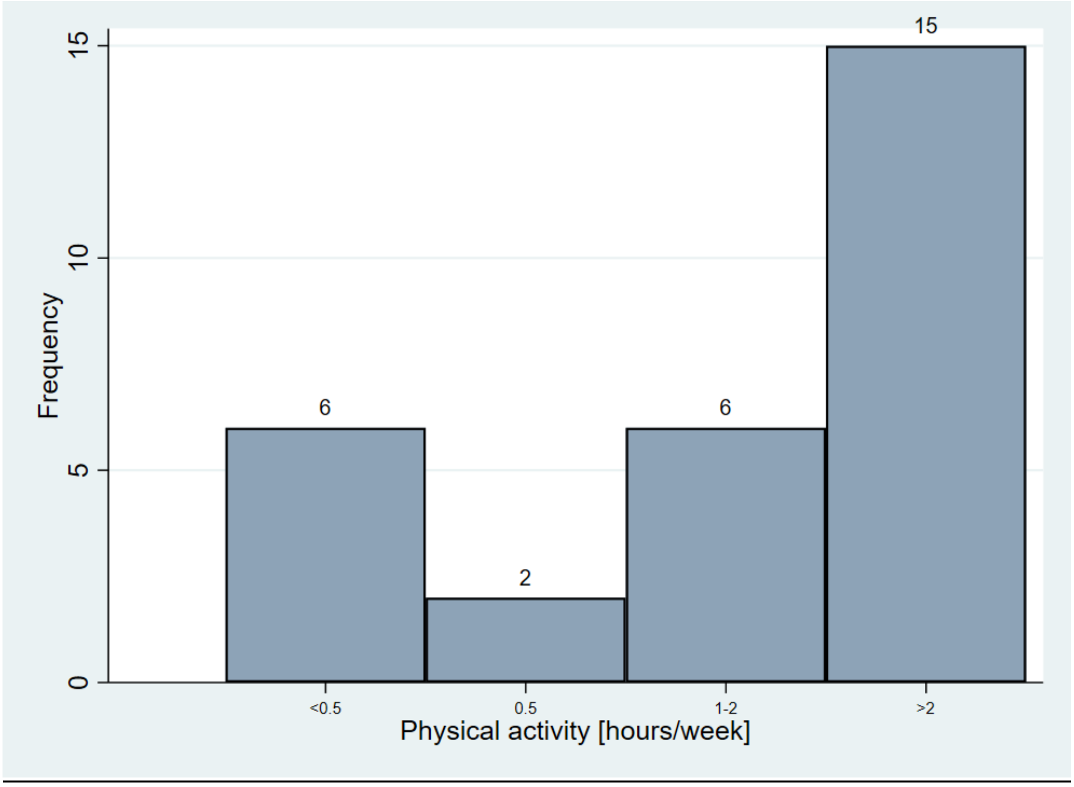

Weight fluctuations

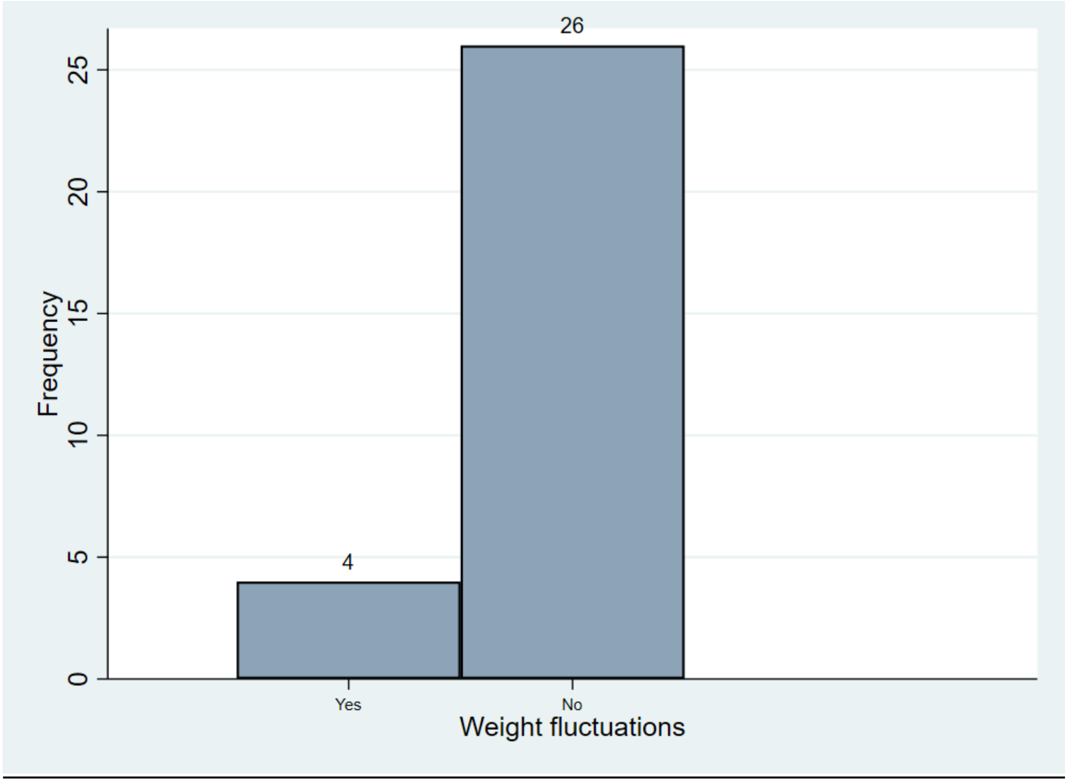

First diet

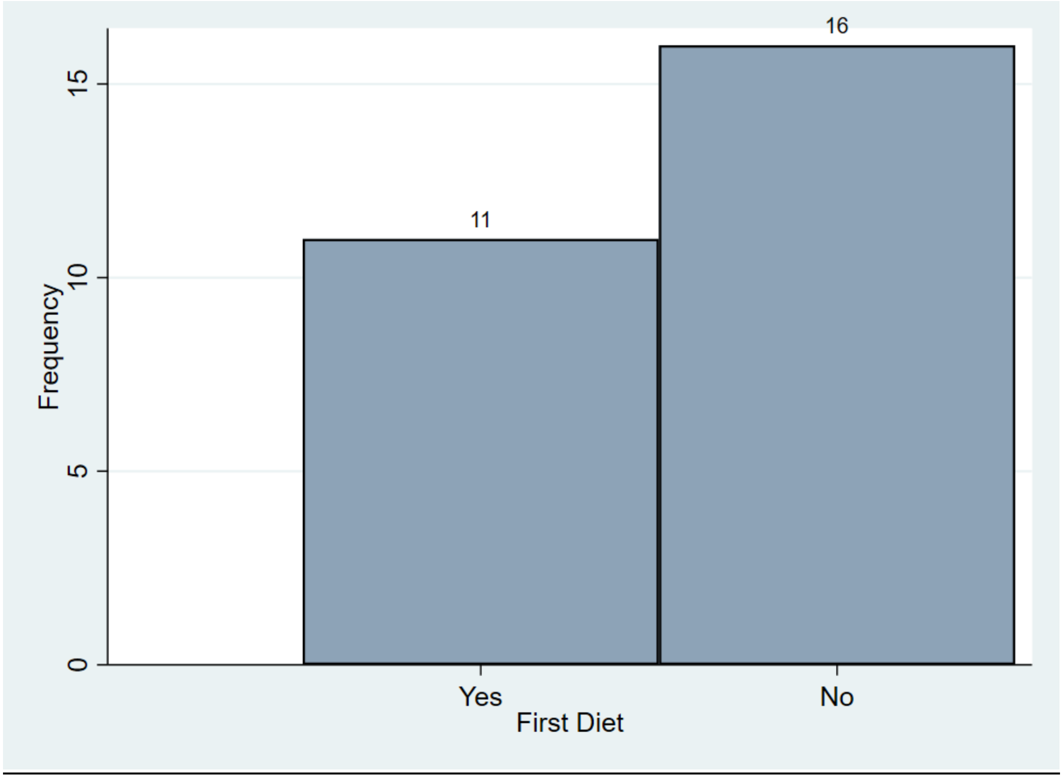

Supplement: Supplementary file 1 [file nutrients-14-04216-s001.zip › nutrients-1857255-supplementary.pdf]
